# Safety of carbon-ion radiotherapy for prostate cancer after rectal surgery: a single-institution retrospective study

**DOI:** 10.1093/jrr/rrag038

**Published:** 2026-05-18

**Authors:** Tomoki Yamaguchi, Atsushi Okato, Masaru Wakatsuki, Kosei Miura, Hirokazu Makishima, Mio Nakajima, Tomokazu Sazuka, Shinichi Sakamoto, Kayoko Ohnishi, Tomohiko Ichikawa, Hitoshi Ishikawa

**Affiliations:** QST Hospital, National Institutes for Quantum Science and Technology, 4-9-1 Anagawa, Chiba-shi, Chiba, 263-8555, Japan; QST Hospital, National Institutes for Quantum Science and Technology, 4-9-1 Anagawa, Chiba-shi, Chiba, 263-8555, Japan; QST Hospital, National Institutes for Quantum Science and Technology, 4-9-1 Anagawa, Chiba-shi, Chiba, 263-8555, Japan; QST Hospital, National Institutes for Quantum Science and Technology, 4-9-1 Anagawa, Chiba-shi, Chiba, 263-8555, Japan; QST Hospital, National Institutes for Quantum Science and Technology, 4-9-1 Anagawa, Chiba-shi, Chiba, 263-8555, Japan; QST Hospital, National Institutes for Quantum Science and Technology, 4-9-1 Anagawa, Chiba-shi, Chiba, 263-8555, Japan; Department of Urology, Graduate School of Medicine, Chiba University, 1-8-1 Inohana, Chuo-ku, Chiba-shi, Chiba, 260-8677, Japan; Department of Urology, Graduate School of Medicine, Chiba University, 1-8-1 Inohana, Chuo-ku, Chiba-shi, Chiba, 260-8677, Japan; Department of Radiology, School of Medicine, International University of Health and Welfare, 852 Hatakeda, Narita-shi, Chiba, 286-8520, Japan; Department of Urology, Funabashi Central Hospital, 6-13-10 Kaijin, Funabashi-shi, Chiba, 273-8556, Japan; QST Hospital, National Institutes for Quantum Science and Technology, 4-9-1 Anagawa, Chiba-shi, Chiba, 263-8555, Japan

**Keywords:** Prostate cancer, C-ion RT, rectal surgery, safety, rectal bleeding, toxicity

## Abstract

Carbon-ion radiotherapy (C-ion RT) is effective in treating prostate cancer. However, the safety of this therapy in patients with a history of rectal surgery remains unclear. This study aimed to confirm the feasibility and efficacy of C-ion RT in patients with prostate cancer after rectal surgery. This study comprised 33 consecutive patients with prostate cancer who underwent C-ion RT at our institute from October 1995 to May 2024 with a history of rectal surgery for their colorectal cancers. The prescribed doses were 63.0 Gy [relative biological effectiveness (RBE)] in 20 fractions (fr), 57.6 Gy (RBE) /16 fr, and 51.6 Gy (RBE) or 54.0 Gy (RBE) /12 fr. The Common Terminology Criteria for Adverse Events, version 5.0, was used to assess toxicity. Risk factors for rectal bleeding, including patient and treatment characteristics, and dosimetric parameters were statistically analyzed. The median patient age and observation period were 70 years and 53.1 months, respectively. Late grade 1 and 2 gastrointestinal toxicities were observed in six (18.2%) and one (3.0%) patients, respectively. Late grade 1 gastrointestinal toxicities included rectal bleeding, and late grade 2 gastrointestinal toxicities included faecal incontinence. The development of late rectal bleeding was significantly associated with the duration from surgery to radiation (*P* = 0.040), especially <5 years (*P* = 0.011), and rectal Dmean (*P* = 0.018). C-ion RT is safe for patients with prostate cancer after rectal surgery, and its toxicity is manageable. A shorter time to radiation from rectal surgery, especially <5 years, and rectal Dmean are risk factors for late rectal bleeding.

## INTRODUCTION

In 2022, prostate and colorectal cancers were the second and third most prevalent types of male cancer, respectively, and constituted the third and fifth leading causes of cancer-related mortality amongst the global male population [1]. Amongst colorectal cancers, 40.8% of men have rectal cancer; therefore, there are often opportunities to consider treatment strategies for patients with synchronous or metachronous prostate cancer with a history of rectal surgery [2, 3]. As the standard treatment for rectal or sigmoid cancer is surgical resection, radical surgery is commonly performed.

Localized prostate cancer is treated primarily through surgery or radiotherapy (RT). Although robot-assisted radical prostatectomy is the preferred surgical method for prostate cancer treatment [4–7], it is unsuitable for patients with a history of rectal surgery for rectal or sigmoid cancer because of adhesions [8]. RT may be an alternative for such patients, but several studies have indicated an association between previous abdominal or pelvic surgical procedures and instances of rectal bleeding [9–11]. Furthermore, few studies have demonstrated the feasibility of definitive RT after rectal surgery [12].

Particle beam therapy is an RT technique that can be categorized as proton beam therapy and carbon-ion RT (C-ion RT). C-ion RT exhibits unique biological and physical properties in RT [13–15]. C-ion RT for prostate cancer produces favourable outcomes in terms of toxicity and may therefore be safe even for patients with a history of rectal surgery [16–20]. Takakusagi *et al*. [20] showed important results about the safety of C-ion RT at Kanagawa Cancer Center. Their study was an excellent first step, but the number of patients was limited (*n* = 13) and the follow-up period was relatively short. Also, we still need more information about dose constraints for the rectum. Therefore, to better understand their results, we conducted a study including a larger number of patients with a longer follow-up. The aim of this study was to find the risk factors for rectal complications more clearly.

## MATERIALS AND METHODS

### Patients

This study comprised patients with prostate cancer who received C-ion RT at our hospital between April 1995 and May 2024 and had a history of rectal surgery for cancer of the rectum or sigmoid colon before C-ion RT. The following criteria were used to determine eligibility: (i) prostate cancer diagnosed based on histopathological analysis, (ii) cT1bN0M0 to cT4 (excluding rectal invasion) N0M0 according to the 7th Union for International Cancer Control classification, (iii) age > 20 years, performance status of 0–2, (iv) history of surgery for rectal or sigmoid cancer before the start of C-ion RT and (vi) rectal anastomosis within or near the irradiation field for C-ion RT. Clinical data were collected in May 2025. The need for written informed consent was waived because of the retrospective observational nature of this study; however, all participants or their relatives had the opportunity to opt out. This study was approved by the Institutional Review Board of National Institutes for Quantum Science and Technology (approval number: N25–006).

### Androgen deprivation therapy

The use of androgen deprivation therapy (ADT) in C-ion RT has been described previously [16, 21–24]. Briefly, in the intermediate- and high-risk groups, patients received ADT combined with C-ion RT. The administration of neoadjuvant ADT was conducted for 2–6 months. The duration of adjuvant ADT was 6 months for intermediate-risk patients and > 24–36 months for high-risk patients. The utilization of ADT exhibited no variation in accordance with the fractionation regimen.

### Carbon-ion radiotherapy

C-ion RT was performed by the protocol described previously [16, 21–24]. C-ion RT was administered to the prostate gland and seminal vesicles once daily for 4 days a week. For dose calculation, the relative biological effectiveness value for C-ion RT was ~3.0 at the distal portion of the spread-out Bragg peak, based on previous studies. Radiation doses were expressed in Gy (relative biological effectiveness-weighted dose based on the modified microdosimetric kinetic model [25]). The present study used the following dose fractionation schemes: 63.0 Gy/20 fractions, 57.6 Gy/16 fractions and 51.6 Gy and 54.0 Gy/12 fractions. The dose regimens administered to the patients in this study initially consisted of 63 Gy in 20 fractions, with some cases receiving 57.6 Gy in 16 fractions. The 57.6 Gy/16 Fr regimen was adopted as the standard from 2008, followed by a transition to 51.6 Gy in 12 fractions in 2013. Since 2022, the dose has been updated to 54.0 Gy in 12 fractions for all subsequent cases. In 2012, the irradiation method transitioned from passive scattering to scanning delivery. The clinical target volume (CTV) comprised the prostate and all or part of the seminal vesicles, whilst pelvic lymph nodes were excluded. Initially, the planning target volume (PTV) margins were 10 mm anteriorly/laterally and 5 mm dorsally/cranially/caudally. Since 2020, the anterior margin has been modified to 7 mm.

The rectum was contoured 1 cm below and 1 cm above the PTV, with a thickness of 2 mm. Rectal anastomosis was contoured using rectal anastomosis clips.

### Patient follow-up

Patients were followed up at 2–6-month intervals in the first year and 6–12-month intervals thereafter. Acute and late toxicities caused by RT were scored in all patients during RT and follow-up. Toxicity was evaluated using the Common Terminology Criteria for Adverse Events version 5.0. The manifestation of toxicity within 3 months of C-ion RT was classified as acute, whereas any occurrence of toxicity after that period was designated as late. The most severe toxicity grade was designated as the final toxicity grade. The observation period and occurrence of each event were derived from the date of the C-ion RT.

### Statistical analyses

The Kaplan–Meier algorithm was utilized to estimate the cumulative incidence of late rectal bleeding from the commencement of C-ion RT to the occurrence of late rectal bleeding or the most recent follow-up. Categorical variables were compared using Fisher’s exact test (two-tailed). Continuous data were compared using the Mann–Whitney U test because of the non-normal distribution of variables. *P* values <0.05 were considered significant. All statistical analyses were performed using R version 4.5.0.

## RESULTS

### Patient parameters

During the observation period from 1995 to 2024, 33 patients whose anastomoses of rectal surgery were located below the upper edge of the irradiation field for C-ion RT were eligible for the present study, and patient characteristics are summarized in Table 1. Amongst them, 29 (87.9%) and 3 (9.0%) patients had rectal and sigmoid colon cancers, respectively, and the remaining 3 (9.0%) simultaneously underwent both cancer surgeries. The median age at the time of rectal surgery was 58 (range, 41–71) years, and the median age at the time of C-ion RT was 70 (range, 57–82) years, with a median interval of 8 years between treatments. The details of the National Comprehensive Cancer Network risk classification were as follows: 1 (3.0%) had low risk, 12 (36.4%) had intermediate risk, and 20 (60.6%) had high risk. The median observation period was 52 (range, 8–191) months.

**Table 1:** Patient characteristics (*n* = 33)

Characteristics	*n* (%), median (range)
Follow-up duration, months	53.1 (8.9–194.4)
Age, years	70 (57–82)
NCCN risk classification	
Low	1 (3.0)
Intermediate	12 (36.4)
High	20 (60.6)
Prescribed dose	
63 Gy/20 Fr	1 (3.0)
57.6 Gy/16 Fr	8 (24.2)
51.6 Gy/12 Fr	14 (30.3)
54 Gy/12 Fr	10 (42.4)
Androgen deprivation therapy	
Yes	32 (97.0)
No	1 (3.0)
Time to C-ion RT from rectal surgery, years	8 (2–26)
Time to C-ion RT from rectal surgery was <5 years	
Yes	7 (21.2)
No	26 (78.8)
Time to C-ion RT from rectal surgery was <10 years	
Yes	18 (54.5)
No	15 (45.5)
Diabetes mellitus	
Yes	6 (18.2)
No	27 (81.8)
Anticoagulation therapy	
Yes	3 (9.1)
No	30 (90.9)
Use of gold markers	
Yes	12 (36.4)
No	21 (63.6)
Anastomosis adjacent to PTV	
Yes	20 (60.6)
No	13 (39.4)

NCCN: National Comprehensive Cancer Network; C-ion RT: Carbon ion radiotherapy; Fr: Fraction; Gy: Grey; n: number of patients; PTV: Planning target volume.

### Dose–volume parameters

**Fig. 1:**
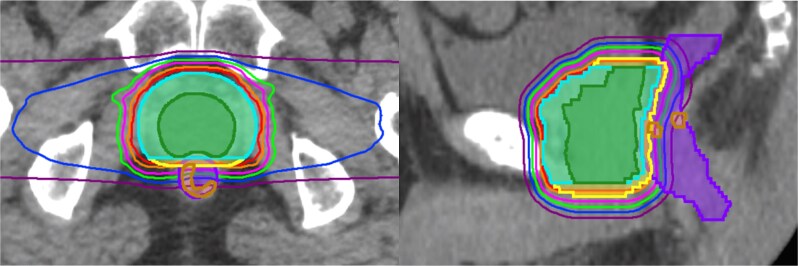
Typical dose distribution of 54Gy/12Fr. The rectum, rectal anastomosis, clinical target volume (CTV), and planning target volumes (PTV1 and PTV2) are delineated.

The prescribed doses were as follows: 63 Gy in 20 fractions (*n* = 1), 57.6 Gy in 16 fractions (*n* = 8), and 51.6 Gy (*n* = 14) and 54 Gy (*n* = 10) in 12 fractions. A typical dose distribution is shown in Fig.  1. The median Dmax and Dmean to the rectal anastomosis were 46.2 Gy (interquartile range [IQR], 1.2–51.6) and 11.6 Gy (IQR 1.15–14.8), respectively. The median dose of rectum D2cc was 45.0 Gy (IQR, 41.5–49.8). The median dose-volumes for the rectum were as follows: V30 = 5.2 cc, V40 = 3.2 cc, and V50 = 0.8 cc. The dosimetric parameters are listed in Table 2.

**Table 2:** Dosimetric parameters of C-ion RT

Structure	Median	Range	IQR
Rectum			
V_10 Gy_ (cc)	9.8	5.5–36.2	8.2–18.6
V_20 Gy_ (cc)	7.3	3.2–22.0	5.7–12.5
V_30 Gy_ (cc)	5.2	1.7–15.6	4.0–8.2
V_40 Gy_ (cc)	3.2	0.6–8.1	2.3–5.7
V_50 Gy_ (cc)	0.8	0.0–4.1	0.2–1.8
D_2 cc_ (Gy)	45.0	27.9–55.6	41.5–49.8
D_6 cc_ (Gy)	26.2	8.4–44.2	18.7–38.4
D_mean_ (Gy)	10.4	0.0–34.8	1.2–14.8
Anastomosis			
V_40 Gy_ (cc)	0.4	0.0–0.4	0.0–0.1
V_50 Gy_ (cc)	0.0	0.0–0.2	0.0–0.0
D_max_ (Gy)	46.2	0.0–57.3	11.2–51.6
D_mean_ (Gy)	11.6	0.0–34.8	1.2–14.8

### Survival outcomes

During the observation period, there were four deaths; however, no deaths from prostate cancer were reported. Furthermore, one patient diagnosed with castration-resistant prostate cancer had an elevated prostate-specific antigen level during hormone therapy, 15 months after C-ion RT. Two patients experienced biochemical relapse at 48 and 57 months after C-ion RT. The former patient experienced a local relapse at 107 months after C-ion RT.

### Acute and late toxicities

**Fig. 2:**
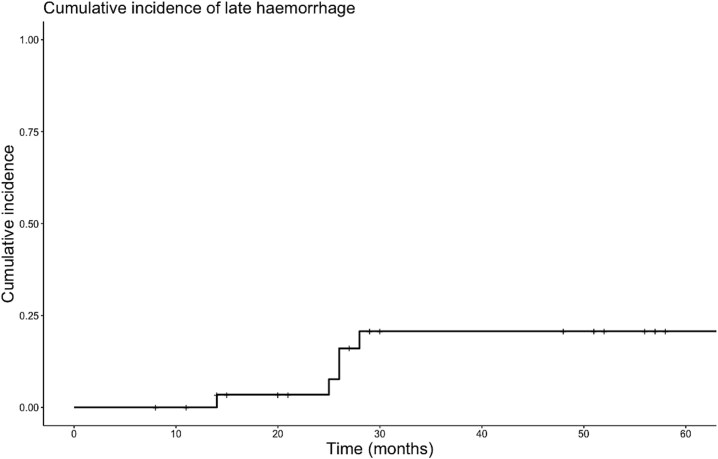
Cumulative incidence of late haemorrhage after carbon ion radiotherapy. The incidence is shown as a function of time. Tick marks indicate censored observations.

Table 3 summarizes the toxicity profiles following C-ion RT. Acute grade 1 and 2 genitourinary toxicities were reported in 12 (36.4%) and 10 (30.3%) cases, respectively. Acute grade 1 gastrointestinal (GI) toxicities were observed in three (9.1%) patients. Acute grade 1 GI toxicities included rectal haemorrhage (*n* = 2) and proctitis (*n* = 1). Late grade 2 genitourinary toxicity was observed in six (18.2%) patients. Late grade 1 and 2 GI toxicities were observed in six (18.2%) and one (3.0%) patients, respectively. Late grade 1 GI toxicities included rectal bleeding, and late grade 2 GI toxicities included faecal incontinence. The cumulative incidence of late rectal bleeding is illustrated in Fig.  2. The duration from rectal surgery to C-ion RT was significantly associated with late rectal bleeding (*P* = 0.040). The median time interval between rectal surgery and C-ion RT in the bleeding group was 4 years, whereas that in the non-bleeding group was 10 years. A period of <5 years from surgery to C-ion RT was significantly associated with bleeding (*P* = 0.027), whereas a period of <10 years was not (*P* = 0.186).

**Table 3:** Toxicities

	Acute			Late		
	Number (%)			Number (%)		
Toxicities	Grade 1	Grade 2	Grade 3 or more	Grade 1	Grade 2	Grade 3 or more
Genitourinary	12 (36.4)	10 (30.3)	0	10 (30.3)	6 (18.2)	0
Gastrointestinal	3 (9.1)	0	0	6 (18.2)	1 (3.0)	0
Others	8 (24.2)	0	0	0	0	0

Neither anticoagulant therapy nor comorbidity of diabetes mellitus (DM) showed a statistically significant association with the occurrence of bleeding (*P* = 1.000 and *P* = 0.563, respectively). Additionally, anastomosis adjacent to the PTV did not show a statistically significant association with late rectal bleeding (*P* = 1.000).

Regarding the dosimetric parameters, Dmean (*P* = 0.018) of the rectum was the only predictive factor for rectal bleeding, whereas D2cc (*P* = 0.084), D6cc (*P* = 0.132), V10Gy (*P* = 0.112), V20Gy (*P* = 0.173), V30Gy (*P* = 0.205), V40Gy (*P* = 0.161) and Dmax (*P* = 0. 072) were not. Anastomotic Dmean (*P* = 0.173), Dmax (*P* = 0.158), V40 Gy (*P* = 0.369) and V50 Gy (*P* = 0.284) were not associated with bleeding. The analysis of factors associated with late rectal bleeding are listed in Table 4.

**Table 4:** The analysis of factors associated with late rectal bleeding

Factors	*P*-value	Risk Ratio (for categorical variables)	Confidence intervals (for categorical variables)
Follow-up duration	0.056		
Age, years	0.544		
Time to C-ion RT from rectal surgery	0.040		
Time to C-ion RT from rectal surgery was <5 years	0.027	7.429	1.694–32.57
Time to C-ion RT from rectal surgery was <10 years	0.186	4.167	0.545–31.878
Diabetes mellitus	0.563	0.308	0.02–4.836
Anticoagulation therapy	1.000	0.596	0.041–8.707
Anastomosis adjacent to PTV	1.000	1.300	0.277–6.107
Anastomosis adjacent to PTV + Diabetes mellitus	1.000	0.596	0.041–8.707
Rectum V_10 Gy_	0.112		
Rectum V_20 Gy_	0.173		
Rectum V_30 Gy_	0.205		
Rectum V_40 Gy_	0.161		
Rectum V_50 Gy_	0.129		
Rectum D_2 cc_	0.084		
Rectum D_6 cc_	0.132		
Rectum D_mean_	0.018		
Anastomosis V_40 Gy_	0.369		
Anastomosis V_50 Gy_	0.284		
Anastomosis D_max_	0.173		
Anastomosis D_mean_	0.158		

## DISCUSSION

To our knowledge, this is the largest report confirming the feasibility of definitive RT for patients with prostate cancer with a history of rectal surgery and the first report evaluating the risk factors for rectal bleeding using dose–volume histogram analysis. There was no grade 3 or more GI toxicity. One patient experienced late grade 2 GI toxicity, which manifested as faecal incontinence but not rectal bleeding. There have been reports of grade 2 faecal incontinence after RT for prostate cancer [9]. However, the age of the patient in our study was >80 years, and he experienced cerebral infarction immediately before faecal incontinence. Cerebral infarction may have affected faecal incontinence, although the influence of C-ion RT cannot be ruled out.

Some studies using three-dimensional conformal radiation therapy have indicated a possible link between a history of abdominal or pelvic surgery and late gastrointestinal toxicity [9–11]. In a previous report of C-ion RT in 13 patients with prostate cancer who had a history of rectal surgery, rectal bleeding occurred in 15.4% of the patients, but there was no grade 2 or severe GI toxicity [25]. In this study, grade 1 rectal bleeding was observed in 18.2% of the patients, but no grade 2 bleeding was observed. These results suggest that C-ion RT is acceptable for patients with prostate cancer with a history of rectal surgery. Conversely, a recent Japanese study evaluated the safety of intensity-modulated radiation therapy (IMRT) in 20 patients with prostate cancer after rectal cancer surgery [12]. In the study, the irradiation doses to the rectum were restricted up to 70 Gy with a conventional dose fractionation, and there was no grade ≥ 2 late GI toxicity. However, grade 1 late GI toxicity occurred in 25% of the patients, which was slightly higher than that in C-ion RT studies, although the comparison of toxicity profiles is uncertain because of the small sample sizes in all studies.

In the statistical analysis for evaluating the risk factors for late rectal bleeding after C-ion RT, the interval time from rectal surgery to C-ion RT, especially a period <5 years, and the Dmean value of the rectum were associated with the occurrence of bleeding. We previously reported that anticoagulation therapy significantly increased the risk of rectal bleeding following 20-fraction C-ion RT for patients with prostate cancer without a history of rectal surgery [26]. However, rectal bleeding did not show a statistically significant association in the present study, mainly because of the small sample size. Takakusagi *et al*. reported that rectal bleeding after C-ion RT frequently occurred when patients with a history of rectal surgery had DM and a rectal anastomosis adjacent to the PTV [20]. However, no statistically significant association was observed between these factors and rectal bleeding in the present study, although the incidence of grade 1 rectal bleeding in both studies was similar. The probable reasons for the discrepancy between the two studies may be a difference in the severity of DM, such as the utilization of insulin by the patient [27].

Our study suggests that the duration from surgery to C-ion RT and intervals of <5 years were risk factors for rectal bleeding. The results support the favourable outcomes obtained from a previous Japanese IMRT study of patients with prostate cancer with a history of rectal surgery [28, 29]. They treated 20 patients who had undergone rectal surgery >5 years before IMRT; grade 1 and grade 2 late GI toxicities were observed in 25% and 0% of the patients, respectively. Rectal surgery worsened blood flow at the rectal anastomosis [28, 30]. Following hemicolectomy, documented reports of ischemic changes suggest the potential influence of venous insufficiency [29, 31]. Accordingly, from the early postoperative phase to several years later, patients may remain at an increased risk of radiation-induced bleeding, potentially driven by circulatory impairment and venous outflow obstruction. They also reported that 70 Gy with conventional fractionated IMRT might be the appropriate limit for the maximum dose for rectal anastomosis. Regarding C-ion RT, Takakusagi *et al.* [32] and our study suggest that anastomosis adjacent to the PTV is not associated with GI toxicity, and anastomoses Dmax, Dmean, V40Gy and V50Gy did not show a significant association with rectal bleeding in the present study. It is possible that a significant reduction in the irradiated doses and volumes to the rectum by C-ion RT could increase the tolerable doses to the anastomosed and normal rectum. Notably, the median V40 of the anastomosis was only 0.4 cc and the V30, V40 and V50 of the rectum were 5.2 cc, 3.2 cc and 0.8 cc, respectively.

Our study revealed that only the rectal Dmean, a markedly low dose, was significantly associated with the development of rectal bleeding. In our previous study, the V50% of the prescribed dose (33 Gy), an intermediate dose, was significantly associated with rectal bleeding. These inconsistent results at the same institution may be due to the postoperative vulnerability of the rectum to fibrosis, scarring or ischemia after rectal surgery [32]. In addition, Ono *et al*. from Yamagata University reported that rectal volumes irradiated with low doses, such as V10 Gy and V20 Gy, correlated with the occurrence of rectal bleeding after C-ion RT for prostate cancer [33]. Rectal volumes irradiated at low to intermediate dose levels in C-ion RT were significantly limited in C-ion RT for prostate cancer, and not at high doses; however, a relatively low dose may cause problematic bleeding. As Huang *et al.* [34] reported that the Dmean stereotactic body radiation therapy and C-ion RT for prostate cancer were 13.87 ± 5.39 and 5.26 ± 4.64 (*P* < 0.001), respectively, C-ion RT may be more suitable than X-ray RT in post-rectal surgery cases. Furthermore, the IMRT study by Zhang *et al.* [12] did not include patients within 5 years after rectal surgery who had a high risk for rectal bleeding in the present study, and late GI toxicities occurred more frequently in their study than in ours. Although the backgrounds were not balanced and the number of cases in both studies was small, these results support our assumption that C-ion RT is an appropriate curative treatment for patients with prostate cancer with a history of rectal surgery.

This study has some limitations. This study was conducted in a single-hospital retrospective study with a small sample size and a short observation period. Additionally, this study did not establish specific dosage constraints for rectal anastomosis. Moreover, the impact of prior chemotherapy remains unclear. As this was a retrospective study and the medical records provided by the referring institutions were incomplete, detailed records of chemotherapy regimens were missing in many cases. It was hard to assess whether chemotherapy-induced microvascular damage was a confounding factor. Furthermore, variations exist in prescription doses and doses per fraction. Further analysis involving a larger patient group and a longer observation period is required to determine the safety of C-ion RT for prostate cancer following rectal surgery.

In conclusion, this study aimed to assess the safety of C-ion RT for prostate cancer after rectal cancer surgery. These findings indicated that the toxicity levels were acceptable. A shorter time to radiation following rectal surgery, especially <5 years, and rectal Dmean are risk factors for late rectal bleeding.

## Presentation at a conference

Yamaguchi T, Okato A, Miura K, *et al*. Safety of carbon-ion radiotherapy for prostate cancer after rectal surgery: A single-institution retrospective study. Presented at: The 38th Annual Meeting of the Japanese Society for Radiation Oncology; November 27–29, 2025.
